# Demographic characteristics associated with West Nile virus neuroinvasive disease – A retrospective study on the wider European area 2006–2021

**DOI:** 10.1371/journal.pone.0292187

**Published:** 2023-09-28

**Authors:** Nicola Riccetti, Federico Ferraccioli, Augusto Fasano, Nikolaos I. Stilianakis

**Affiliations:** 1 European Commission, Joint Research Centre (JRC), Ispra (VA), Italy; 2 Department of Biometry and Epidemiology, University of Erlangen-Nuremberg, Erlangen, Germany; University of Pécs: Pecsi Tudomanyegyetem, HUNGARY

## Abstract

**Background:**

With a case-fatality-risk ranging from 3.0 to >20.0% and life-long sequelae, West Nile neuroinvasive disease (WNND) is the most dangerous outcome of West Nile virus (WNV) infection in humans. As no specific prophylaxis nor therapy is available for these infections, focus is on preventive strategies. We aimed to find variables associated with WNND diagnosis, hospitalisation or death, to identify high-risk sub-groups of the population, on whom to concentrate these strategies.

**Methods:**

We used data from The European Surveillance System–TESSy, provided by National Public Health Authorities, and released by the European Centre for Disease Prevention and Control (ECDC). In two Firth-penalised logistic regression models, we considered age, sex, clinical criteria, epidemiological link to other cases (epi-link), calendar year, and season as potential associated variables. In one model we considered also the rural/urban classification of the place of infection (RUC), while in the other the specific reporting country.

**Findings:**

Among confirmed West Nile Virus cases, 2,916 WNND cases were registered, of which 2,081 (71.4%), and 383 (13.1%) resulted in the hospitalisation and death of the patient, respectively. Calendar year, RUC/country, age, sex, clinical criteria, and epi-link were associated with WNND diagnosis. Hospitalisation was associated with calendar year and RUC/country; whereas death was associated with age, sex and country.

**Interpretation:**

Our results support previous findings on WNND associated variables (most notably age and sex); while by observing the whole population of WNND cases in the considered area and period, they also allow for stronger generalizations, conversely to the majority of previous studies, which used sample populations.

## Introduction

West Nile virus (WNV) is considered as one of the worldwide leading causes of infectious encephalitis [1] and the causative agent of 1.4% of all central nervous system diseases in humans [2]. WNV is maintained in an enzootic cycle mostly between mosquitoes and birds, with other vertebrates–including humans–as occasional spillover and dead-end hosts [1, 3].

The disease resulting from WNV infection in humans is considered to be mostly asymptomatic (∼ 80.0%) or to present mild symptomatology with fever, headache, and fatigue (West Nile fever [WNF], ∼ 20.0%) [1, 4]. Less than 1% of WNV human cases develop in a neurological disease (West Nile neuroinvasive disease [WNND]), which usually presents in three clinical forms, or a combination of them: encephalitis (WNE, 50.0–71.0% of WNND cases), meningitis (WNM, 15.0–35.0%), or acute flaccid paralysis (AFP, 3.0–19.0%) [1, 5–8]. In all three forms, the majority of patients presents with neurological signs or deficit, alongside the aforementioned symptoms [9–11]. Albeit in non-complicated cases of WNF and WNM full recovery is normally expected, often symptoms might persist in the longer term, especially following WNE [2–4, 6, 12–18]. An elevated proportion of WNND patients might require hospitalisation (≥ 80.0%), regardless of the clinical form or the disease [16]; and almost two patients in three (61.0%) require admission to the intensive care unit (ICU) [19]. Moreover, WNND presents with a case fatality risk (CFR) ranging from 3.0 to >20.0% [2, 5, 8, 9, 20, 21], with elderly patients and patients with comorbidities being at higher risk of succumbing to the disease [3, 12, 16, 21]. Several studies have considered the clinical picture, the severity of the outcome, and the CFR in WNND to be influenced by demographic characteristics of the individual (most notably age and sex), as well as clinical characteristics (e.g. form of the disease, and comorbidities such as diabetes, hypertension, and renal failure) [1, 3, 6, 8, 11, 16, 20, 22–24].

Considering that, to date, no vaccination nor specific therapy is available for the disease resulting from WNV infections in humans [3, 5], preventive actions are the only strategy to contain the disease. Hence, understanding the characteristics associated with WNND development, hospitalisation and death could allow focusing specific public health interventions on target groups of the population with higher risk. However, most of the studies on this topic consider a single or few countries, often outside of the wider European area. Moreover, these studies often present with small study sample size, which might hinder generalisations of the results.

For these reasons, we aimed at exploring the potential demographic characteristics associated with developing, being hospitalised, and succumbing to WNND, using a dataset comprehensive of the whole population of WNND cases in the wider European area, over a 15-year period.

Our results could largely confirm previous studies’ findings, while at the same time allowing for larger generalizations. More in detail, we observed an association between WNND diagnosis and calendar year, place of infection, age, sex, and having being exposed to mosquitoes bites in an endemic area or to an infected transfusion. Hospitalisation was associated with year and place of infection, whereas death was associated with age, sex and the specific country.

## Materials and methods

### Data collection

We used WNV human cases from The European Surveillance System (TESSy), provided by Albania, Austria, Belgium, Bosnia and Herzegovina, Bulgaria, Croatia, Cyprus, Czechia, France, Germany, Greece, Hungary, Ireland, Italy, Kosovo, Montenegro, the Netherlands, North Macedonia, Portugal, Romania, Serbia, Slovakia, Slovenia, Spain, Sweden, Türkiye, and the United Kingdom, and released by the European Centre for Disease Prevention and Control (ECDC).

To use a justified sample, firstly we selected only WNV confirmed cases, defined as any case with at least one of the following: (a) isolation of WNV from blood/cerebrospinal fluid (CSF), (b) detection of WNV nucleic acid in blood/CSF, (c) WNV specific antibody response (immunoglobulin M [IgM]) in CSF, or (d) presenting IgM high titre and detection of WNV immunoglobulin G (IgG) and confirmation by neutralisation [25]. Secondly, among these confirmed cases, we considered only the ones listed with WNND clinical manifestation [25].

#### Variables operationalisation

Age (*continuous*), sex (female/male/unknown), calendar year (*continuous*), and country name (of the reporting country), were included in their original form. Calendar month was grouped into seasons (autumn [October, November, and December]/winter [January, February, and March]/spring [April, May, and June]/summer [July, August, and September]/missing [if no information was available regarding the month of registration]). Clinical criteria, considered “yes” if the individual presented with at least one between fever, encephalitis, or meningitis, and epidemiological link to other cases (epi-link), considered “yes” if the individual was exposed to mosquito bites in WNV endemic areas or to human-to-human transmission (e.g. transfusion), were both recoded to “yes” and “no/unknown” (originally yes/no/unknown). To account for the difference in access to care between rural and urban regions, the code of the place of infection in the TESSy dataset was joined to the European Statistical Office (EUROSTAT) list of Nomenclature of Territorial Units for Statistics level 3 (NUTS-3). A categorical variable representing the rural/urban classification of the place of infection (RUC) was created and coded as predominantly urban if at least 80.0% of the population lives in urban clusters (defined as continuous grid cells of 1km^2^ with population density ≥300 inhabitants/km^2^ and a minimum population of 5,000 inhabitants); intermediate if between 50.0% and 79.0% of the population lives in urban clusters; and predominantly rural if at least 50.0% of the population lives in grid cells that are not urban centres [26]. For the endpoints, three dichotomous variables (yes/no) for WNND, and hospitalisation/death due to the disease were created. Finally, we created two continuous variables counting the days since the onset of symptoms and the diagnosis/hospitalisation.

## Statistical analysis

### Descriptive statistics

WNND confirmed cases, hospitalisations, and deaths were reported stratified by year and RUC/country. The mean and standard deviation (sd) values of age in years and of the time between the onset of symptoms and diagnosis/hospitalisation in days were reported by RUC/country. To avoid biased estimations due to outliers, we filtered time between the onset of symptoms and diagnosis/hospitalisation to have absolute values smaller than 180 days. Similarly, the sex distribution of WNND cases, hospitalised and dead individuals, and the proportion of hospitalisations and deaths due to the disease were reported by RUC/country.

### Regression analysis

We developed two regression models: in the first, RUC (ref.: predominantly rural), age (*continuous*), sex (ref.: female), were considered as potentially associated variables, whereas calendar year (*continuous*), season (ref: Summer), clinical criteria (ref.: no), and epi-link (ref.: no), as potential confounders. In the second, the RUC was substituted with the name of the country (ref: Greece, as the country with highest cases count after Romania, and without complete separation between hospitalised and non-hospitalised). All the variables considered potentially associated with the outcomes of interest (WNND case, hospitalisation and death) were derived from previous publication on characteristics of WNND [16, 17, 21, 24, 27]. Three logistic regressions were fitted for each set of potentially associated variables, one for each of the outcomes of interest. When considering death as outcome of interest, we included also hospitalisation (ref: no) as a potential associated variable. To avoid estimates influenced by sparse-data bias, we utilised Firth penalisation method [28, 29]. We reported the results of these models in terms of odds ratio (OR) and relative 95% confidence intervals (CI), obtained as the exponential values of the estimate and relative confidence interval. More in detail, as Firth penalization results in confidence intervals with approximated coverage, also their exponential values should be considered as such. Furthermore, as with Firth penalisation *p*-values are not available, in the result section we reported the variables for which the estimated confidence interval does not contain 1.

As the time between the onset of symptoms and hospitalisation/diagnosis presented with numerous missing values, these variables were excluded from the models.

All calculations were conducted in R (version: 4.0.3).

## Results

### Descriptive analysis

#### WNND confirmed cases

*N* = 7,913 WNV cases were registered in the wider European area between 2006 and 2021. Among these, 5,869 (74.1%) were considered as confirmed cases, of which 2,916 (49.7%) were registered as WNND. These latter 2,916 cases were considered for this study. *N* = 608 (20.8%) of the considered cases presented with predominantly rural RUC, 925 (31.7%) with intermediate RUC, and 574 (19.7%) with predominantly urban RUC; no information was available regarding the RUC for the remaining records (27.8%). Romania (633), Greece (579), Serbia (576), and Italy (568) were the countries with the highest prevalence. The proportion of imported cases ranged between 0% in several countries and 100% (out of four cases) in Sweden.

All records presented as either male or female. In the overall population, 1,090 cases (37.4%) were female. When stratifying by RUC, female case proportion ranged from 35.1% (*n* = 325) to 39.6% (*n* = 241) within individuals with intermediate and rural RUC, respectively. Among the single countries female proportion of cases ranged from 0% in Albania and Portugal (out of eight and one case, respectively) to 54.5% (*n* = 6) in Bulgaria.

Overall, the average age among WNND cases was 64.9 years (sd = 16.8). The average age ranged between 63.3 (sd = 15.9) and 67.0 years (sd = 16.9) among cases with missing and predominantly urban RUC, respectively. Among the single countries, the average age ranged from 48.1 years (sd = 19.3) in the Netherlands to 74.5 years (sd = 6.4) in Sweden.

Nine records were eliminated for presenting with time between onset of symptoms and diagnosis and/or time between onset of symptoms and hospitalization greater than 180 days in absolute value. Among these records, four presented with time between onset of symptoms and diagnosis greater than 180 days in absolute value, while four presented time between onset of symptoms and hospitalisation greater than 180 days in absolute value. One record presented with both variables greater than 180 days in absolute value. These nine records were mostly male (seven), reported from Romania and Türkiye (three each, while the remaining three were one each in France, Italy, and Serbia), and mostly with predominantly urban or missing RUC (three each, while the remaining three presented twice with an intermediate RUC and once with a predominantly rural RUC). All these nine records were eliminated. The average time between onset of symptoms and diagnosis in the remaining records ranged between 13.5 days (sd = 7.5, median = 12.0, interquartile range [IQR] = 7.0) to 15.3 days (sd = 16.7, median = 11.0, IQR = 8.0) among cases with missing/predominantly urban RUC, respectively. Among the single countries, it ranged between 9.0 days (sd = 3.0, median = 9.0, IQR = 3.0) in North Macedonia and 55.8 days (sd = 38.1, median = 61.0, IQR = 73.8) in Türkiye (Table 1 and Supplementary table S1 in S1 File).

**Table 1:** West Nile neuroinvasive disease (WNND) cases among West Nile virus (WNV) confirmed cases by year and rural/urban classification of the place of infection (predominantly rural [Rur]/intermediate [Int]/predominantly urban [Urb] and missing [*NaN*]) as well as International Organization for Standardization (ISO) Country code of the reporting country. Included are the proportion of sex (expressed as absolute and relative frequencies of female cases), and of imported cases (absolute and relative frequencies), mean age in years (and standard deviation [sd]) and mean (and sd) of the time between onset of symptoms and diagnosis (OD) expressed in days.

Year	Rural/Urban*				Country ISO Code**																					Total (Mean/sd)
	Rur	Int	Urb	*NaN*	AL	AT	BG	HR	CY	CZ	FR	DE	GR	HU	IT	XK	NL	MK	PT	RO	RS	SI	ES	SE	TR	
2006	2	1	0	*0*	0	0	0	0	0	0	0	0	0	1	0	0	0	0	0	2	0	0	0	0	0	**3**
2007	3	4	0	*1*	0	0	0	0	0	0	0	0	0	4	0	0	0	0	0	4	0	0	0	0	0	**8**
2008	1	15	1	*3*	0	0	0	0	0	0	0	0	0	18	0	0	0	0	0	2	0	0	0	0	0	**20**
2009	1	4	0	*2*	0	0	0	0	0	0	0	0	0	5	0	0	0	0	0	2	0	0	0	0	0	**7**
2010	61	72	50	*7*	0	0	0	0	0	0	1	0	103	17	5	0	0	0	0	52	0	0	2	0	10	**190**
2011	24	24	24	*13*	7	0	0	0	0	0	1	0	48	4	14	0	0	0	0	10	0	0	0	0	1	**85**
2012	10	26	52	*23*	0	1	0	6	0	0	3	0	47	8	28	4	0	0	0	14	0	0	0	0	0	**111**
2013	34	53	24	*28*	0	0	0	20	0	0	1	0	36	12	45	2	0	0	0	22	0	1	0	0	0	**139**
2014	25	30	3	*64*	0	0	0	1	0	0	0	0	12	2	21	4	0	0	0	23	59	0	0	0	0	**122**
2015	14	39	13	*35*	0	1	0	1	0	0	0	0	0	12	38	1	0	0	1	19	28	0	0	0	0	**101**
2016	40	72	29	*56*	0	1	0	2	1	0	3	0	0	17	41	0	1	0	0	85	41	0	3	0	2	**197**
2017	46	36	27	*57*	0	2	0	5	0	0	0	0	10	8	26	2	0	0	0	64	45	0	0	0	4	**166**
2018	205	371	224	*469*	1	6	6	52	1	4	6	3	164	113	244	12	1	0	0	267	362	4	1	4	18	**1269**
2019	73	93	45	*28*	0	1	4	0	17	1	2	4	77	21	24	0	0	3	0	58	23	0	0	0	4	**239**
2020	50	46	62	*3*	0	0	1	0	0	0	0	4	59	1	47	0	6	0	0	3	0	0	40	0	0	**161**
2021	19	39	20	*20*	0	1	0	0	0	0	1	2	23	6	35	0	0	0	0	6	18	0	6	0	0	**98**
**Sum**	**608**	**925**	**574**	***809***	**8**	**13**	**11**	**87**	**19**	**5**	**18**	**13**	**579**	**249**	**568**	**25**	**8**	**3**	**1**	**633**	**576**	**5**	**52**	**4**	**39**	**2916**
Sex *n* (%)	241 (39.6)	325 (35.1)	212 (36.9)	*312 (38*.*6)*	0 (0)	5 (38.5)	6 (54.5)	33 (37.9)	2 (10.5)	1 (20.0)	5 (27.8)	2 (15.4)	226 (39.0)	77 (30.9)	194 (34.2)	11 (44.0)	2 (25.0)	1 (33.3)	0 (0)	256 (40.4)	231 (40.1)	1 (20.0)	23 (44.2)	1 (25.0)	13 (33.3)	**1090 (37.4)**
Imported *n* (%)	1 (0.2)	3 (0.3)	2 (0.3)	*52 (6*.*4)*	0 (0)	3 (23.1)	1 (9.1)	5 (5.7)	0 (0)	1 (20.0)	13 (72.2)	4 (30.8)	5 (0.9)	9 (3.6)	7 (1.2)	1 (4.0)	2 (25.0)	0 (0)	0 (0)	1 (0.2)	0 (0)	0 (0)	2 (3.8)	4 (100)	0 (0)	**58 (2.0)**
Age mean, sd	65.7, 17.3	64.6, 17.1	67.0, 16.9	*63*.*3*, *15*.*9*	59.1 18.5	64.8, 17.4	63.3, 9.5	64.9, 14.8	74.0, 11.6	59.0, 14.5	63.0, 12.4	69.7, 13.3	68.0, 16.6	58.7, 17.1	70.5, 14.2	65.2, 17.8	48.1, 19.3	69.0, 11.5	71.0, ‐‐	61.5, 18.0	63.9, 15.3	68.2, 6.4	60.4, 19.5	74.5, 6.4	52.6, 24.5	**(64.9,** **16.8)**
OD mean, sd	14.6, 14.0	14.2, 11.8	15.3, 16.7	*13*.*5*, *7*.*5*	10.1, 2.3	15.1, 18.6	15.3, 9.6	20.8, 7.0	*NaN*	50.0, ‐‐	20.4, 18.8	30.3, 18.0	10.6, 5.8	14.4, 10.9	*NaN*	12.8, 8.2	51.5, 32.6	9.0, 3.0	34.0, ‐‐	15.6, 12.1	13.0, 5.8	44.4, 67.0	12.7, 11.4	25.5, 12.2	55.8, 38.1	**(14.3, 12.5)**

(*) Rural/urban classification of the place of infection is coded as (a) predominantly urban if at least 80% of the population lived in urban clusters (defined as continuous grid cells of 1km2 with population density ≥300 inhabitants/km2 and a minimum population of 5,000 inhabitants); intermediate if between 50% and 79% of the population lives in urban clusters; and predominantly rural if at least 50% of the population lives in grid cells that are not urban centres [26].

(**) List of ISO Country codes: AL ‐ Albania, AT ‐ Austria, BG ‐ Bulgaria, HR ‐ Croatia, CY ‐ Cyprus, CZ ‐ Czechia, FR ‐ France, DE ‐ Germany, GR ‐ Greece, HU ‐ Hungary, IT ‐ Italy, XK ‐ Kosovo, NL ‐ The Netherlands, MK ‐ North Macedonia, PT–Portugal, RO ‐ Romania, RS ‐ Serbia, SI ‐ Slovenia, ES ‐ Spain, SE–Sweden, TR–Türkiye.

#### Hospitalisation due to WNND

*N* = 2,081 cases (71.4% of the total) were hospitalised. The proportion of all cases hospitalised ranged from 53.5% to 91.8% among cases with intermediate and missing RUC, respectively. Among the single countries, the proportion of all cases hospitalised ranged between 65.5% in Croatia to 100% in most other countries.

Overall, 38.3% (*n* = 797) of hospitalised cases were females. The female proportion of hospitalised cases ranged between 35.6% (*n* = 176) and 39.8% (*n* = 296) among cases with intermediate and missing RUC, respectively. Among the single countries, it ranged between 0% in Albania and Portugal (out of eight and one case, respectively) to 54.5% in Bulgaria (*n* = 6).

Average age of hospitalised cases ranged between 60.6 years (sd = 17.6) and 66.1 years (sd = 17.9) among cases with intermediate RUC and cases with predominantly urban RUC, respectively. Among the single countries, the average age ranged between 48.1 years (sd = 19.3) in the Netherlands and 74.5 years (sd = 6.4) in Sweden.

The average time between onset of symptoms and diagnosis in hospitalised cases ranged between 13.5 days (sd = 7.5) and 16.1 days (sd = 17.4) among cases with missing and predominantly urban RUC, respectively.

Average time between onset of symptoms and hospitalisation ranged between 4.2 days (sd = 4.4) and 4.7 days (sd = 4.2) among cases with predominantly rural and missing RUC, respectively (Table 2 and Supplementary table S2 in S1 File).

**Table 2:** Hospitalisations due to human West Nile neuroinvasive disease (WNND) cases among West Nile virus (WNV) confirmed cases by year and rural/urban classification of the place of infection (predominantly rural [Rur]/intermediate [Int]/predominantly urban [Urb] and missing [*NaN*]) as well as International Organization for Standardization (ISO) Country code of the reporting country. Included are the proportion of cases hospitalised out of all WNND cases (Prop), of sex (expressed as absolute and relative frequencies of female cases), and of imported cases (absolute and relative frequencies), mean age in years and mean (and sd) of time between onset of symptoms and diagnosis (OD) and hospitalisation (OH), both in days.

Year	Rural/Urban*				Country ISO Code**																			Total (Mean, sd)
	Rur	Int	Urb	*NaN*	AL	AT	BG	HR	CY	CZ	FR	GR	HU	XK	NL	MK	PT	RO	RS	SI	ES	SE	TR	
2006	1	1	0	*0*	0	0	0	0	0	0	0	0	0	0	0	0	0	2	0	0	0	0	0	**2**
2007	3	4	0	*1*	0	0	0	0	0	0	0	0	4	0	0	0	0	4	0	0	0	0	0	**8**
2008	1	15	1	*3*	0	0	0	0	0	0	0	0	18	0	0	0	0	2	0	0	0	0	0	**20**
2009	1	4	0	*1*	0	0	0	0	0	0	0	0	4	0	0	0	0	2	0	0	0	0	0	**6**
2010	20	40	13	*4*	0	0	0	0	0	0	1	0	12	0	0	0	0	52	0	0	2	0	10	**77**
2011	2	6	6	*8*	7	0	0	0	0	0	1	0	3	0	0	0	0	10	0	0	0	0	1	**22**
2012	4	8	8	*10*	0	1	0	0	0	0	3	0	8	4	0	0	0	14	0	0	0	0	0	**30**
2013	22	23	22	*7*	0	0	0	0	0	0	1	36	12	2	0	0	0	22	0	1	0	0	0	**74**
2014	23	10	3	*63*	0	0	0	0	0	0	0	12	1	4	0	0	0	23	59	0	0	0	0	**99**
2015	10	11	7	*33*	0	1	0	0	0	0	0	0	11	1	0	0	1	19	28	0	0	0	0	**60**
2016	29	45	28	*52*	0	1	0	0	1	0	3	0	17	0	1	0	0	85	41	0	3	0	2	**154**
2017	41	21	24	*54*	0	2	0	5	0	0	0	10	8	2	0	0	0	64	45	0	0	0	4	**140**
2018	163	208	182	*461*	1	3	6	52	1	4	6	164	109	12	1	0	0	267	362	4	1	4	17	**1014**
2019	68	78	38	*26*	0	1	4	0	17	1	2	77	20	0	0	3	0	58	23	0	0	0	4	**210**
2020	46	15	48	*0*	0	0	1	0	0	0	0	59	1	0	6	0	0	3	0	0	39	0	0	**109**
2021	14	6	15	*20*	0	1	0	0	0	0	1	23	0	0	0	0	0	6	18	0	6	0	0	**55**
**Sum**	**448**	**495**	**395**	***743***	**8**	**10**	**11**	**57**	**19**	**5**	**18**	**381**	**228**	**25**	**8**	**3**	**1**	**633**	**576**	**5**	**51**	**4**	**38**	**2081**
Prop (%)	73.7	53.5	68.8	*91*.*8*	100	76.9	100	65.5	100	100	100	65.8	91.6	100	100	100	100	100	100	100	98.1	100	97.4	**71.4**
Sex *n* (%)	175 (39.1)	176 (35.6)	150 (38.0)	*296 (39*.*8)*	0 (0)	5 (50.0)	6 (54.5)	26 (45.6)	2 (10.5)	1 (20.0)	5 (27.8)	144 (37.8)	70 (30.7)	11 (44.0)	2 (25.0)	1 (33.3)	0 (0)	256 (40.4)	231 (40.1)	1 (20.0)	22 (43.1)	1 (25.0)	13 (34.2)	**797 (38.3)**
Imported *n* (%)	1 (0.2)	3 (0.6)	2 (0.5)	*35 (4*.*7)*	0 (0)	3 (30.0)	1 (9.1)	5 (8.8)	0 (0)	1 (20.0)	13 (72.2)	2 (0.5)	6 (2.6)	1 (4.0)	2 (25.0)	0 (0)	0 (0)	1 (0.2)	0 (0)	0 (0)	4 (3.9)	4 (100)	0 (0)	**41** **(2.0)**
Age mean, sd	64.3, 17.9	60.6, 17.6	66.1, 17.9	*63*.*4*, *15*.*7*	59.1, 18.5	64.3, 19.5	63.3, 9.5	65.9, 14.8	74.0, 11.6	59.0, 14.5	63.0, 12.4	69.1, 16.4	59.2, 16.9	65.2, 17.8	48.1, 19.3	69.0, 11.5	71.0, ‐‐	61.5, 18.0	63.9, 15.3	68.2, 6.4	60.4, 19.7	74.5, 6.4	51.9, 24.5	**(63.4, 17.2)**
OD mean, sd	15.2, 14.7	14.6, 11.8	16.1, 17.4	*13*.*5*, *7*.*5*	10.1, 2.3	8.2, 7.0	15.3, 9.6	20.8, 7.0	*NaN*	50.0, ‐‐	20.4, 18.8	11.3, 6.0	13.8, 9.9	12.8, 8.2	51.5, 32.6	9.0, 3.0	34.0, ‐‐	15.6, 12.1	13.0, 5.8	44.4, 67.0	12.7, 11.5	25.5, 12.2	54.7, 38.0	**(14.7, 12.7)**
OH mean, sd	4.2, 4.4	4.5, 6.0	4.3, 6.4	*4*.*7*, *4*.*2*	3.6, 1.3	7.0, ‐‐	5.7, 5.1	5.0, ‐‐	3.9, 6.4	10.2, 6.7	*NaN*	4.6, 6.4	5.2, 6.4	5.1, 3.6	*NaN*	4.0, 1.7	6.0, ‐‐	3.9, 4.3	4.7, 4.3	5.0, 1.4	4.7, 3.5	3.0, 2.2	2.4, 10.0	**(4.4, 5.2)**

(*) Rural/urban classification of the place of infection is coded as (a) predominantly urban if at least 80% of the population lived in urban clusters (defined as continuous grid cells of 1km2 with population density ≥300 inhabitants/km2 and a minimum population of 5,000 inhabitants); intermediate if between 50% and 79% of the population lives in urban clusters; and predominantly rural if at least 50% of the population lives in grid cells that are not urban centres [26].

(**) List of ISO Country codes: AL ‐ Albania, AT ‐ Austria, BG ‐ Bulgaria, HR ‐ Croatia, CY ‐ Cyprus, CZ ‐ Czechia, FR ‐ France, GR ‐ Greece, HU ‐ Hungary, XK ‐ Kosovo, NL ‐ The Netherlands, MK ‐ North Macedonia, PT–Portugal, RO ‐ Romania, RS ‐ Serbia, SI ‐ Slovenia, ES ‐ Spain, SE–Sweden, TR–Türkiye.

#### Fatalities due to WNND

*N* = 383 fatalities (13.1% of the total) were recorded. The overall proportion of fatalities was 13.1% and 18.4% among confirmed and hospitalised cases, respectively. The proportion of confirmed cases succumbing to the disease ranged between 9.0% to 17.6% among cases with missing and predominantly rural RUC, respectively. Similarly, the proportion of hospitalised cases that succumbed to the disease ranged from 9.8% to 23.9% among cases with missing and predominantly rural RUC, respectively. When considering the single countries, the proportion of fatalities among confirmed and hospitalised cases ranged from 5.3% to 33.3% for both values in Cyprus and North Macedonia, respectively.

Overall, 33.4% (*n* = 128) of fatalities were females. The proportion of female fatalities ranged between 29.8% (*n* = 34) and 37.4% (*n* = 40) among cases with intermediate and predominantly rural RUC, respectively.

Average age ranged between 73.6 years (sd = 13.4) and 78.3 years (sd = 9.3) among cases with missing and predominantly urban RUC, respectively. In the single countries, it ranged between 68.0 years (sd = 24.0) in Spain and 88.0 years (only one case) in Cyprus.

Average time between onset of symptoms and diagnosis ranged between 12.3 days (sd = 5.2) and 16.3 days (sd = 18.3) among individuals with predominantly rural and predominantly urban RUC, respectively. Average time between onset of symptoms and hospitalisation ranged between 3.3 days (sd = 8.1) and 3.8 days (sd = 4.0) in individuals with intermediate and missing RUC (Table 3 and Supplementary table S3 in S1 File).

**Table 3:** Deaths due to West Nile neuroinvasive disease (WNND) cases among West Nile virus (WNV) confirmed cases by year and rural/urban classification of the place of infection (predominantly rural [Rur]/intermediate [Int]/predominantly urban [Urb] and missing [*NaN*]) as well as the International Organization for Standardization (ISO) Country code of the reporting country. Included are the proportion of fatalities out of all WNND cases (Prop C) and out of all hospitalised (Prop H), the proportion of sex (expressed as absolute and relative frequencies of female cases), and of imported cases (absolute and relative frequencies), mean age in years and mean (and sd) of time between onset of symptoms and diagnosis (OD) and hospitalisation (OH), both in days.

Year	Rural/Urban*				Country ISO Code**																Total (Mean, sd)
	Rur	Int	Urb	*NaN*	AL	BG	HR	CY	CZ	DE	GR	HU	IT	XK	MK	RO	RS	ES	SE	TR	
2010	9	12	7	*0*	0	0	0	0	0	0	17	1	0	0	0	5	0	0	0	5	**28**
2011	5	1	3	*2*	0	0	0	0	0	0	7	0	3	0	0	1	0	0	0	0	**11**
2012	2	2	4	*2*	0	0	0	0	0	0	8	0	0	1	0	1	0	0	0	0	**10**
2013	6	0	4	*0*	0	0	0	0	0	0	9	0	1	0	0	0	0	0	0	0	**10**
2014	4	1	1	*9*	0	0	0	0	0	0	5	0	0	1	0	1	8	0	0	0	**15**
2015	1	0	0	*3*	0	0	0	0	0	0	0	0	0	0	0	1	3	0	0	0	**4**
2016	9	8	8	*4*	0	0	0	0	0	0	0	4	2	0	0	19	2	1	0	1	**29**
2017	8	6	6	*5*	0	0	1	0	0	0	2	3	1	1	0	14	2	0	0	1	**25**
2018	33	63	38	*42*	1	1	4	0	1	1	31	10	49	3	0	38	33	0	1	3	**176**
2019	14	14	8	*1*	0	0	0	1	0	0	21	1	4	0	1	8	1	0	0	0	**37**
2020	15	6	9	*2*	0	1	0	0	0	0	16	0	7	0	0	1	0	7	0	0	**32**
2021	1	1	1	*3*	0	0	0	0	0	0	1	0	0	0	0	1	3	1	0	0	**6**
**Sum**	**107**	**114**	**89**	***73***	**1**	**2**	**5**	**1**	**1**	**1**	**117**	**19**	**67**	**6**	**1**	**90**	**52**	**9**	**1**	**10**	**383**
Prop C (%)	17.6	12.3	15.5	*9*.*0*	12.5	18.2	5.7	5.3	20.0	7.7	20.2	7.6	11.8	24.0	33.3	14.2	9.0	17.3	25.0	25.6	13.1
Prop H (%)	23.9	23.0	22.5	*9*.*8*	12.5	18.2	8.8	5.3	20.0	‐‐	30.7	8.3	‐‐	24.0	33.3	14.2	9.0	17.6	25.0	26.3	18.4
Sex *n* (%)	40 (37.4)	34 (29.8)	31 (34.8)	*23 (31*.*5)*	0 (0)	0 (0)	2 (40.0)	0 (0)	1 (100)	0 (0)	38 (32.5)	3 (15.8)	22 (32.8)	4 (66.7)	0 (0)	36 (40.0)	16 (30.8)	3 (33.3)	1 (100)	2 (20.0)	**128** **(33.4)**
Imported *n* (%)	0 (0)	0 (0)	0 (0)	*4* *(5*.*5)*	0 (0)	0 (0)	0 (0)	0 (0)	0 (0)	1 (100)	0 (0)	0 (0)	1 (1.5)	0 (0)	0 (0)	0 (0)	0 (0)	1 (11.1)	1 (100)	0 (0)	**4** **(1.0)**
Age mean, sd	76.2, 8.8	75.2, 9.4	78.3, 9.3	*73*.*6*, *13*.*4*	73.0, ‐‐	69.5, 4.9	76.4, 4.4	88.0, ‐‐	71.0, ‐‐	78.0, ‐‐	78.5, 7.0	70.9, 12.3	77.0, 9.9	69.0, 11.8	81.0, ‐‐	74.9, 10.3	75.6, 10.4	68.0, 24.0	81.0, ‐‐	68.5, 12.2	**(75.9, 10.2)**
OD mean, sd	12.3, 5.2	13.5, 11.7	16.3, 18.3	*13*.*7*, *9*.*5*	*NaN*	26.0, ‐‐	23.0, ‐‐	*NaN*	50.0, ‐‐	*NaN*	10.6, 4.5	10.8, 7.0	*NaN*	9.0, 6.6	9.0, ‐‐	14.7, 7.6	13.1, 4.9	19.6, 21.8	19.0, ‐‐	46.4, 43.0	**(13.9, 12.0)**
OH mean, sd	3.7, 4.5	3.3, 8.1	3.7, 3.5	*3*.*8*, *4*.*0*	*NaN*	9.5, 9.2	*NaN*	1.0, ‐‐	6.0, ‐‐	*NaN*	3.8, 3.6	3.5, 4.8	*NaN*	2.7, 1.2	5.0, ‐‐	3.9, 5.3	3.9, 4.3	4.0, 3.0	6.0, ‐‐	-3.4, 14.4	**(3.6, 5.2)**

(*) Rural/urban classification of the place of infection is coded as (a) predominantly urban if at least 80% of the population lived in urban clusters (defined as continuous grid cells of 1km2 with population density ≥300 inhabitants/km2 and a minimum population of 5,000 inhabitants); intermediate if between 50% and 79% of the population lives in urban clusters; and predominantly rural if at least 50% of the population lives in grid cells that are not urban centres [26].

(**) List of ISO Country codes: AL ‐ Albania, BG ‐ Bulgaria, HR ‐ Croatia, CY ‐ Cyprus, CZ ‐ Czechia, FR ‐ France, DE ‐ Germany, GR ‐ Greece, HU ‐ Hungary, IT ‐ Italy, XK ‐ Kosovo, MK ‐ North Macedonia, RO ‐ Romania, RS ‐ Serbia, ES ‐ Spain, SE–Sweden, TR–Türkiye

### Regression analysis

As the classification of all clinical criteria is missing, data from Germany were excluded from the regression analysis. Similarly, as the classification of all hospitalisations is “*null*”, cases from Italy were excluded from regressions considering hospitalisation as outcome/potentially associated variable.

Calendar year was associated with WNND cases and hospitalisation for both the considered sets of variables (OR = 0.91; 95% CI [0.88, 0.94], and OR = 1.73; 95% CI [1.61, 1.87], respectively for the first model and OR = 1.05; 95% CI [1.01, 1.08], and OR = 1.64; 95% CI [1.51, 2.11], respectively for the second model) (Tables 4 and 5).

**Table 4:** Odds ratio (OR) and 95% confidence intervals (CI) of the variables for which the association with West Nile neuroinvasive disease (WNND) diagnosis, hospitalisation (Hosp) and death was investigated using firth-penalized logistic regression model considering rural/urban classification of place of infection (*). The number of records entering each model (*n*) is reported.

Covariates	WNND (*n* = 2,887)			Hosp (*n* = 1,552)			Death (*n* = 2,099)		
	OR	95% CI		OR	95% CI		OR	95% CI	
		Lower	Upper		Lower	Upper		Lower	Upper
Year	0.91	0.88	0.94	1.73	1.61	1.87	1.00	0.95	1.06
Season (ref. Summer)									
Winter	†	†	†	†	†	†	†	†	†
Spring	2.29	0.78	7.90	1.55	0.26	10.74	1.11	0.11	6.00
Autumn	1.74	0.93	3.52	3.49^††^	1.05^††^	14.80^††^	1.44	0.83	2.42
Rural/urban (ref. Predominantly rural)									
Intermediate	0.42	0.31	0.56	2.64	1.50	4.69	0.77	0.54	1.10
Predominantly urban	0.70	0.50	0.98	0.63	0.36	1.09	0.77	0.54	1.09
Age	1.04	1.03	1.04	0.99	0.98	1.00	1.09	1.07	1.10
Sex (ref. Female)									
Male	1.27	1.03	1.57	1.33	0.84	2.10	1.44	1.06	1.96
Clinical criteria (ref. No)									
Yes	129.72^††^	43.86^††^	635.94^††^	†	†	†	†	†	†
Epidemiologically linked (ref. No)									
Yes	47.77	24.20	110.97	247.68^††^	63.80^††^	2243.26^††^	0.98	0.71	1.35
Hospitalisation (ref No)									
Yes	‐‐	‐‐	‐‐	‐‐	‐‐	‐‐	1.18	0.66	2.10

(*)Rural/urban classification of the place of infection is coded as (a) predominantly urban if at least 80% of the population lived in urban clusters (defined as continuous grid cells of 1km2 with population density ≥300 inhabitants/km2 and a minimum population of 5,000 inhabitants); intermediate if between 50% and 79% of the population lives in urban clusters; and predominantly rural if at least 50% of the population lives in grid cells that are not urban centres [26]

†: Estimate not shown because of potential unreliability arising from complete separation

^††^: Estimate shown although potentially unreliable due to near complete separation (defined as one level ≤5% of the other)

**Table 5:** Odds ratio (OR) and 95% confidence intervals (CI) of the variables for which the association with West Nile neuroinvasive disease (WNND) confirmed diagnosis, hospitalisation (Hosp) and death was investigated using firth-penalised logistic regression model considering the single reporting countries. The number of records entering each model (*n*) is reported.

Covariates	WNND (*n* = 3,881)			Hosp (*n* = 2,283)			Death (*n* = 2,283)		
	OR	95% CI		OR	95% CI		OR	95% CI	
		Lower	Upper		Lower	Upper		Lower	Upper
Year	1.05	1.01	1.08	1.64	1.51	2.11	1.01	0.96	1.07
Season (ref. Summer)									
Winter	0.57	0.04	9.43	†	†	†	†	†	†
Spring	1.23	0.48	3.51	0.98	0.03	93.33	1.42	0.25	5.52
Autumn	1.09	0.67	1.81	1.83	0.20	140.29	1.13	0.72	1.71
Countries (ref. Greece)									
Albania	0.24	0.05	1.13	†	†	†	0.58	0.03	8.98
Austria	0.06	0.03	0.12	0.45	0.02	30.67	†	†	†
Belgium	†	†	†	‐‐	‐‐	‐‐	‐‐	‐‐	‐‐
Bulgaria	0.64	0.17	3.68	†	†	†	1.87	0.32	7.80
Croatia	†	†	†	0.78	< .01	>1000.00	0.44^††^	0.02^††^	7.41^††^
Cyprus	1.28	0.29	12.73	†	†	†	0.18	0.02	0.79
Czechia	0.12	0.03	0.49	†	†	†	2.52	0.21	18.20
France	0.29	0.09	0.92	†	†	†	†	†	†
Hungary	0.59	0.40	0.87	14.22	4.51	115.67	0.54	0.31	0.92
Italy	0.09	0.07	0.12	‐‐	‐‐	‐‐	‐‐	‐‐	‐‐
Kosovo	1.29	0.38	6.87	†	†	†	1.65	0.57	4.26
Netherlands	0.74	0.16	4.65	†	†	†	†	†	†
North Macedonia	†	†	†	†	†	†	2.71	0.20	25.49
Portugal	†	†	†	†	†	†	†	†	†
Romania	75.63^††^	15.78^††^	504.49^††^	†	†	†	0.43	0.05	3.68
Serbia	15.97^††^	4.27^††^	64.18^††^	†	†	†	0.23	0.03	1.98
Slovenia	†	†	†	†	†	†	†	†	†
Spain	7.29^††^	1.26^††^	79.72^††^	4.61^††^	< .01^††^	>1000.00^††^	0.63	0.07	5.46
Sweden	1.12	0.07	26.20	†	†	†	0.58	0.03	9.61
Türkiye	0.35	0.19	0.66	30.86^††^	2.14^††^	>1000.00^††^	3.15	1.27	7.58
United Kingdom	†	†	†	‐‐	‐‐	‐‐	‐‐	‐‐	‐‐
Age	1.04	1.03	1.04	1.00	0.97	1.03	1.08	1.07	1.10
Sex (ref. Female)									
Male	1.28	1.05	1.57	1.03	0.34	3.06	1.50	1.15	1.98
Clinical criteria (ref. No)									
Yes	†	†	†	21.31	< .01	>1000.00	†	†	†
Epidemiologically linked (ref. No)									
Yes	0.64	0.21	1.89	0.91^††^	< .01^††^	>1000.00^††^	2.05	0.25	15.70
Hospitalisation (ref No)									
Yes	‐‐	‐‐		‐‐	‐‐	‐‐	1.20	0.67	2.19

†: Estimate not shown because of potential unreliability arising from complete separation

^††^: Estimate shown although potentially unreliable due to near complete separation (defined as one level ≤5% of the other)

Similarly, age and sex were associated with WNND cases and death for both the considered sets of variables (OR = 1.04; 95% CI [1.03, 1.04], and OR = 1.09; 95% CI [1.07, 1.10], respectively for age in the first model, and OR = 1.04; 95% CI [1.03, 1.04], and OR = 1.08; 95% CI [1.07, 1.10], respectively for age in the second model, as well as OR = 1.27; 95% CI [1.03, 1.57], and OR = 1.44; 95% CI [1.06, 1.96], respectively for male vs female in the first model, and OR = 1.29; 95% CI [1.05, 1.57], and OR = 1.50; 95% CI [1.15, 1.98], respectively for male vs female in the second model) (Tables 4 and 5).

RUC was associated with WNND cases and hospitalisation for the first set of variables (OR = 0.42; 95% CI [0.31, 0.56]; and OR = 0.70; 95% CI [0.50, 0.98] for intermediate and predominantly urban areas vs predominantly rural, respectively, and OR = 2.64; 95% CI [1.50, 4.69] for intermediate vs predominantly rural) (Tables 4 and 5).

Country was associated with WNND cases, hospitalisation and death (OR = 0.06; 95% CI [0.03, 0.12]; OR = 0.12; 95% CI [0.03, 0.49], OR = 0.29; 95% CI [0.09, 0.92], OR = 0.59; 95% CI [0.40, 0.87], OR = 0.09; 95% CI [0.07, 0.12], and OR = 0.35; 95% CI [0.19, 0.66] for Austria, Czechia, France, Hungary, Italy, and Türkiye, respectively, vs Greece for WNND cases; OR = 14.22; 95% CI [4.52, 115.05] for Hungary vs Greece for hospitalisation; and OR = 0.18; 95% CI [0.02, 0.79], OR = 0.54; 95% CI [0.31, 0.92], OR = 3.15; 95% CI [1.27, 7.58] for Cyprus, Hungary, and Türkiye, respectively, vs Greece for death) (Tables 4 and 5).

## Discussion

Using the whole population of WNV confirmed cases registered as WNND in the wider European area between 2006–2021, we aimed at exploring certain potential demographic characteristics associated with developing, being hospitalised for or succumbing to WNND. Previous studies observed an association between certain demographic characteristics of the patients (e.g. age, sex, and place of infection) and the aforementioned outcomes [1, 3, 6, 8, 11, 16, 22–24]. In our study, we could mostly confirm these results.

We observed an average age among WNND cases of 64.9 years (sd = 16.8), and we found older age to be positively associated with developing WNND, compared to other forms of the disease. Similarly, Bode et al. [27] observed that patients ≥50 years of age had almost a three times higher risk to develop WNE compared to WNF; Carson et al. [24] and Danis et al. [21] reported a 16- and 50-fold higher risk to develop WNND among patients >65 years of age, as well as an increase of the incidence of WNND in patients ≥80 years of age, compared to WNF, respectively.

Moreover, in our descriptive results about two-thirds (62.6%) of cases were males, and, as for the odds to develop WNND, we estimated a 1.27 odds ratio for males compared to females. In terms of percentage change this means that–according to our results–the odds of developing WNND for males are 27% higher than the odds for females. Carson et al. [24] reported that WNV-infected males have almost one-third higher risk to develop WNND, compared to WNV-infected females. Multiple studies in the review by Yeung et al. [16] reported significant associations between the male sex and a higher risk of developing WNND/WNE compared to WNF/asymptomatic disease or to the general population, albeit the level of significance of these results is often labile.

As for the odds to develop WNND, we estimated a 0.42 and 0.70 odds ratio for individuals with intermediate and predominantly urban RUC, compared to the ones with predominantly rural RUC. Differently from before–according to our results–the odds of developing WNND for individuals with intermediate and urban RUC are 58% and 30% lower, respectively, than the odds for individuals with rural RUC. Danis et al. [21] reported a two-fold higher risk of developing WNND among people residing in rural areas, compared to people residing in urban ones. This association is often considered controversial: although true that WNV vectors are influenced by the surrounding environment, at the same time different vectors have adapted to different environments (e.g., *Cx*. *tarsalis* and *Cx*. *pipiens* in rural and urban/semi-urban environments, respectively). Nevertheless, when interpreting our results, the elevated number of missing values for RUC in our dataset should be considered. In addition, although after filtering for unknown/missing place of residence/infection the discrepancies between these two variables accounted only for a small proportion of WNND records (7.4%), when interpreting our results, it is also important to note that the RUC derived from the place of infection and not of residence, hence it represent better the relative chances of getting infected in certain areas, rather than getting infected if residing in certain others.

In our results, almost three confirmed WNND cases in four (71.4%) were hospitalised. Both Koch et al. [11] and Yeung et al. [16] reported a proportion of more than four WNND patients in five to require hospitalisation.

As hospitalisation is mostly needed among WNM cases, which is more common among younger patients, previous studies have questioned the association between older age and the risk of hospitalisation [4]. Hawkes et al. [19] reported that WNND patients <60 years of age were more likely to be admitted to ICU, compared to older patients. Conversely, Ouhoumanne et al. [20] observed a higher proportion of WNE patients–who should thus be averagely older–admitted to ICU compared to WNM/WNF patients, with no difference in the proportions of overall hospitalisation. Lindsey et al. [23] and Koch et al. [11] reported a significant association between older age and hospitalisation, and between being >65 years of age and developing severe neurological symptoms after hospital admission, respectively. In our results the average age was lower among hospitalised, compared to both WNND confirmed cases and deaths, nevertheless age was not associated with hospitalisation in both our models.

We observed a CFR of 13.1% and 18.4% among overall and hospitalised cases, respectively. Descriptively, CFR was higher among cases with predominantly rural RUC, both among overall cases and among only hospitalised cases. The large majority of the studies included in the review by Yeung et al. [16] reported an association between older age and WNND-induced mortality. Similarly, Hawkes et al. [19] and Bode et al. [27] reported an association between older age and in-hospital mortality among ICU WNND patients, and mortality among WNE patients, respectively. Our results confirm these observations: mean age was higher in cases which succumbed to WNND, compared cases and hospitalised (74.9 years vs 64.9 and 63.4, respectively). Moreover, in both our models, age was positively associated with mortality. Popescu et al. [17] reported a statistically significant difference in the average age of WNND cases succumbing to the disease compared to the survivors, and an association between being >75 years and succumbing to the disease [17]. In this study, the totality of the fatalities presented with comorbidities [17]. This aspect could propose an interpretation of our findings, as we do not have information about the clinical status of the patients.

Finally, we observed an association between sex and mortality. Descriptively, a higher proportion of males succumbed to the disease both among confirmed and hospitalised cases (66.6% vs 62.6% and 61.6%, respectively) and, when investigating the chances to succumb to WNND in the regression models, males presented with odds ratios equal to 1.44 and 1.50 in the model with RUC and in the one with the specific countries, respectively, compared to females. Although the higher male proportion in our sample could influence this result, accordance can be found in previous works in Yeung et al. [16].

### Limitations

The dataset used in this study allowed us a stronger generalization of certain results. However, it also presented with limitations, most notably the absence of clinical information on the cases that disallowed us from exploring the role of comorbidities in developing, being hospitalised, and succumbing to WNND. Moreover, we cannot ignore the possible risk of bias represented by the difference in the disease surveillance strategies among the single countries. In fact, it is not difficult to imagine the presence of a bias in WNV diagnosis between countries that have a longer history of WNV, compared to countries with more recent history of WNV. In addition, the same scenario could be present between different years: in fact, as WNV becomes more present every year, diagnosis might be more punctual compared to previous years. This study considers data from the beginning of WNV surveillance, a different level of completeness of the dataset in the different years might be present, with the first years being potentially less complete than the last ones. Finally, differences were present also for the level of completeness of the single variables in the dataset, with the result–in extreme cases–that the coefficient estimation for certain factor levels was limited by complete separation and therefore not interpretable. Although this issue remains confined to the single problematic levels, the non-affected levels should be interpreted as estimates valid for the subsample of cases that do not present complete separation [30]. This study focused on demographic variables associated with WNND cases, hospitalisation and death. Climatic factors, which are known to influence both the population of WNV vectors as well as their ability to transmit the virus were not included. Nevertheless, we considered this aspect not to penalize the analysis, as we observe different countries over several years and thus we preferred to exclude climatic parameters rather than use mean values over large time periods and/or including multiple dummy variables.

### Implications for researchers and policy makers

We aimed to fill the lack of large population studies over a longer time, which could allow for stronger generalizations on variables associated with WNND. Our results could foster further research aiming to disentangle the reasons why demographic characteristics are associated with a different risk of developing WNND or experiencing severe outcomes of the disease. Moreover, the role of the clinical status could be further observed.

Considering the impact on the quality of life of the patients as well as the costs of WNND, policy makers should focus on implementing and improving strategies to prevent WNV infections in humans. Following our results, these strategies could focus on specific sub-groups of the population (e.g. elderly males) or higher risk areas (e.g. rural), perhaps including targeted communication campaigns and building awareness about the disease within rural communities.

## Conclusions

In the wider European area between 2006–2021, 2,916 confirmed WNV cases were registered as WNND, resulting in 2,081 (71.4%), and 383 (13.1%) hospitalisations and deaths, respectively. Year, RUC/country of reporting, age, sex, and having being exposed to mosquitoes bites in an endemic area or to an infected transfusion were associated with developing WNND. Year and RUC/country were associated with hospitalisation, and death was associated with age, sex and the country of infection.

## Supporting information

S1 File (PDF)
