# Supplementary material for: Demographic characteristics associated with West Nile virus neuroinvasive disease – A retrospective study on the wider European area 2006–2021
Source: PLoS One. 2023 Sep 28;18(9):e0292187. doi: 10.1371/journal.pone.0292187 (PMC10538693; doi:10.1371/journal.pone.0292187)
Supplement: S1 File — (PDF) [file pone.0292187.s001.pdf]

Supporting Information

Table S1. Additional information on distribution of the continuous variables age, and onset-to-diagnosis (OD) among human cases of West Nile virus neuroinvasive disease (WNND) by rural/urban classification of the place of infection (Predominantly rural [Rur], intermediate [Int], predominantly urban [Urb], and missing [NaN]) at the European Statistical Office (EUROSTAT) list of Nomenclature of Territorial Units for Statistics level 3 (NUTS-3), as well as by country reported with their ISO Code. Reported are mean values, standard deviations (SD), median values, and Interquartile ranges (IQR)

Covariates		Rural/Urban classification*				Country ISO Code**																				
		Rur	Int	Urb	NaN	AL	AT	BG	HR	CY	CZ	FR	DE	GR	HU	IT	XK	NL	MK	PT	RO	RS	SI	ES	SE	TR
Age	Mean	65.7	64.6	67.0	63.3	59.1	64.8	63.3	64.9	74.0	59.0	63.0	69.7	68.1	58.7	70.5	65.2	48.1	69.0	71.0	61.5	63.9	68.2	60.4	74.5	52.6
	SD	17.3	17.1	16.9	15.9	18.5	17.4	9.5	14.8	11.6	14.5	12.4	13.3	16.6	17.1	14.2	17.8	19.3	11.5		18.0	15.3	6.4	19.5	6.4	24.5
	Median	69.0	69.0	71.0	66.0	66.5	66.0	66.0	68.0	75.0	51.0	65.5	76.0	72.0	63.0	74.0	68.0	47.0	68.0	71.0	65.0	66.0	67.0	64.5	74.0	60.0
OD	Mean	14.6	14.2	15.3	13.5	10.1	15.1	15.3	20.8		50.0	20.4	30.3	10.6	14.4		12.8	51.5	9.0	34.0	15.6	13.0	44.4	12.7	25.5	55.8
	SD	14.0	11.8	16.7	7.5	2.3	18.6	9.6	7.0			18.8	18.0	5.8	10.9		8.2	32.6	3.0		12.1	5.8	67.0	11.4	12.2	38.1
	Median	12.0	11.0	11.0	12.0	10.0	10.5	13.0	22.0		50.0	13.5	22.0	9.0	11.0		10.0	51.5	9.0	34.0	13.0	12.0	19.0	10.0	23.0	61.0
	IQR	7.0	8.5	8.0	7.0	2.0	13.0	16.8	5.0		0.0	16.3	16.5	6.0	9.0		12.0	43.3	3.0	0.0	8.0	7.0	7.0	8.0	13.0	73.8

(*) Rural/urban classification of the place of infection is coded as (a) predominantly urban if at least 80% of the population lived in urban clusters (defined as continuous grid cells of 1km2 with population density ≥300 inhabitants/km2 and a minimum population of 5,000 inhabitants); intermediate if between 50% and 79% of the population lives in urban clusters; and predominantly rural if at least 50% of the population lives in grid cells that are not urban centres [26].
(**) List of ISO Country codes: AL - Albania, AT - Austria, BG - Bulgaria, HR - Croatia, CY - Cyprus, CZ - Czechia, FR - France, GR - Greece, HU - Hungary, IT - Italy, XK - Kosovo, NL - The Netherlands, MK - North Macedonia, PT – Portugal, RO - Romania, RS - Serbia, SI - Slovenia, ES - Spain, SE – Sweden, TR – Türkiye

Table S2. Additional information on distribution of the continuous variables age, onset-to-diagnosis (OD), and onset-to-hospitalisation (OH) among hospitalised cases of West Nile virus neuroinvasive disease (WNND) by rural/urban classification of the place of infection (Predominantly rural [Rur], intermediate [Int], predominantly urban [Urb], and missing [NaN]) at the European Statistical Office (EUROSTAT) list of Nomenclature of Territorial Units for Statistics level 3 (NUTS-3), as well as by country reported with their ISO Code. Reported are mean values, standard deviations (SD), median values, and Interquartile ranges (IQR)

Covariates		Rural/urban classification*				Country ISO Code**																		
		Rur	Int	Urb	NaN	AL	AT	BG	HR	CY	CZ	FR	GR	HU	XK	NL	MK	PT	RO	RS	SI	ES	SE	TR
Age	Mean	64.3	60.6	66.1	63.4	59.1	64.3	63.3	65.9	74.0	59.0	63.0	69.1	59.2	65.2	48.1	69.0	71.0	61.5	63.9	68.2	60.4	74.5	51.9
	SD	17.9	17.6	17.9	15.7	18.5	19.5	9.5	14.8	11.6	14.5	12.4	16.4	16.9	17.8	19.3	11.5		18.0	15.3	6.4	19.7	6.4	24.5
	Median	68.0	65.0	71.0	66.0	66.5	66.5	66.0	68.0	75.0	51.0	65.5	73.0	63.0	68.0	47.0	68.0	71.0	65.0	66.0	67.0	65.0	74.0	58.5
OD	Mean	15.2	14.6	16.1	13.5	10.1	8.2	15.3	20.8		50.0	20.4	11.3	13.8	12.8	51.5	9.0	34.0	15.6	13.0	44.4	12.7	25.5	54.7
	SD	14.7	11.8	17.4	7.5	2.3	7.0	9.6	7.0			18.8	6.0	9.9	8.2	32.6	3.0		12.1	5.8	67.0	11.5	12.2	38.0
	Median	12.0	12.0	12.0	12.0	10.0	8.5	13.0	22.0		50.0	13.5	10.0	11.0	10.0	51.5	9.0	34.0	13.0	12.0	19.0	10.0	23.0	60.0
	IQR	8.0	8.0	8.0	7.0	2.0	12.0	16.8	5.0		0.0	16.3	6.0	9.0	12.0	43.3	3.0	0.0	8.0	7.0	7.0	8.0	13.0	73.0
OH	Mean	4.2	4.5	4.3	4.7	3.6	7.0	5.7	5.0	3.9	10.2		4.6	5.2	5.1		4.0	6.0	3.9	4.7	5.0	4.7	3.0	2.4
	SD	4.4	6.0	6.4	4.2	1.3		5.1		6.4	6.7		6.4	6.4	3.6		1.7		4.3	4.3	1.4	3.5	2.2	10.0
	Median	3.0	3.0	4.0	4.0	4.0	7.0	5.0	5.0	2.5	7.0		4.0	4.0	4.0		5.0	6.0	3.0	4.0	5.0	4.0	2.5	2.0
	IQR	4.0	4.0	4.0	5.0	0.5	0.0	7.0	0.0	2.3	2.0		5.0	5.0	4.0		1.5	0.0	3.0	5.0	1.0	5.0	2.0	4.0

(*) Rural/urban classification of the place of infection is coded as (a) predominantly urban if at least 80% of the population lived in urban clusters (defined as continuous grid cells of 1km2 with population density ≥300 inhabitants/km2 and a minimum population of 5,000 inhabitants); intermediate if between 50% and 79% of the population lives in urban clusters; and predominantly rural if at least 50% of the population lives in grid cells that are not urban centres [26].

(**) List of ISO Country codes: AL - Albania, AT - Austria, BG - Bulgaria, HR - Croatia, CY - Cyprus, CZ - Czechia, FR - France, GR - Greece, HU - Hungary, XK - Kosovo, NL - The Netherlands, MK - North Macedonia, PT – Portugal, RO - Romania, RS - Serbia, SI - Slovenia, ES - Spain, SE – Sweden, TR – Türkiye

Table S3. Additional information on distribution of the continuous variables age, onset-to-diagnosis (OD), and onset-to-hospitalisation (OH) among cases succumbed to West Nile virus neuroinvasive disease (WNND) by rural/urban classification of the place of infection (Predominantly rural [Rur], intermediate [Int], predominantly urban [Urb], and missing [NaN]) at the European Statistical Office (EUROSTAT) list of Nomenclature of Territorial Units for Statistics level 3 (NUTS-3), as well as by country reported with their ISO Code. Reported are mean values, standard deviations (SD), median values, and Interquartile ranges (IQR)

Covariates		Rural/urban classification*				Country ISO Code**													
		Rur	Int	Urb	*NaN*	AL	BG	HR	CY	CZ	GR	HU	XK	MK	RO	RS	ES	SE	TR
Age	Mean	76.2	73.5	78.2	74.0	73.0	69.5	76.4	88.0	71.0	78.5	70.9	69.0	81.0	74.9	75.6	68.0	81.0	68.5
	SD	8.5	9.9	9.7	12.9		4.9	4.4			7.0	12.3	11.8		10.3	10.4	24.0		12.2
	Median	77.0	74.0	79.0	75.5	73.0	69.5	76.0	88.0	71.0	78.0	72.0	70.5	81.0	77.0	76.0	73.0	81.0	68.0
OD	Mean	12.3	13.5	16.3	13.7		26.0	23.0		50.0	10.6	10.8	9.0	9.0	14.7	13.1	19.6	19.0	46.4
	SD	5.2	11.7	18.3	9.5						4.5	7.0	6.6		7.6	4.9	21.8		43.0
	Median	12.0	11.0	12.0	12.0		26.0	23.0		50.0	10.0	9.0	8.0	9.0	15.0	12.0	13.0	19.0	40.5
	IQR	6.0	7.8	8.3	8.0		0.0	0.0		0.0	5.3	3.5	3.0	0.0	6.8	6.3	10.0	0.0	75.8
OH	Mean	3.7	3.3	3.7	3.8		9.5		1.0	6.0	3.8	3.5	2.7	5.0	3.9	3.9	4.0	6.0	-3.4
	SD	4.5	8.1	3.5	4.0		9.2				3.6	4.8	1.2		5.3	4.3	3.0		14.4
	Median	3.0	3.0	3.0	3.0		9.5		1.0	6.0	3.0	2.0	2.5	5.0	3.0	3.0	3.0	6.0	1.0
	IQR	4.8	5.0	2.0	5.0		6.5		0.0	0.0	4.0	3.0	1.8	0.0	4.0	6.0	4.8	0.0	5.0

(*) Rural/urban classification of the place of infection is coded as (a) predominantly urban if at least 80% of the population lived in urban clusters (defined as continuous grid cells of 1km2 with population density ≥300 inhabitants/km2 and a minimum population of 5,000 inhabitants); intermediate if between 50% and 79% of the population lives in urban clusters; and predominantly rural if at least 50% of the population lives in grid cells that are not urban centres [26].

(**) List of ISO Country codes: AL - Albania, AT - Austria, BG - Bulgaria, HR - Croatia, CY - Cyprus, CZ - Czechia, FR - France, GR - Greece, HU - Hungary, XK - Kosovo, NL - The Netherlands, MK - North Macedonia, PT – Portugal, RO - Romania, RS - Serbia, SI - Slovenia, ES - Spain, SE – Sweden, TR – Türkiye
